# Identification of molecular genetic contributants to canine cutaneous mast cell tumour metastasis by global gene expression analysis

**DOI:** 10.1371/journal.pone.0208026

**Published:** 2018-12-19

**Authors:** Kelly Bowlt Blacklock, Zeynep Birand, Deborah Biasoli, Elena Fineberg, Sue Murphy, Debs Flack, Joyce Bass, Stefano Di Palma, Laura Blackwood, Jenny McKay, Trevor Whitbread, Richard Fox, Tom Eve, Stuart Beaver, Mike Starkey

**Affiliations:** 1 Animal Health Trust, Newmarket, Suffolk, United Kingdom; 2 Institute of Veterinary Science, University of Liverpool, Neston, United Kingdom; 3 IDEXX Laboratories, Ltd, Wetherby, United Kingdom; 4 Abbey Veterinary Services, Newton Abbot, United Kingdom; 5 Finn Pathologists, Harleston, United Kingdom; 6 Nationwide Laboratory Services, Poulton-le-Fylde, United Kingdom; Colorado State University, UNITED STATES

## Abstract

Cutaneous mast cell tumours are one of the most common canine cancers. Approximately 25% of the tumours metastasise. Activating *c-kit* mutations are present in about 20% of tumours, but metastases occur in the absence of mutations. Tumour metastasis is associated with significantly diminished survival in spite of adjuvant chemotherapy. Available prognostic tests do not reliably predict whether a tumour will metastasise. In this study we compared the global expression profiles of 20 primary cutaneous mast cell tumours that metastasised with those of 20 primary tumours that did not metastasise. The objective was to identify genes associated with mast cell tumour metastatic progression that may represent targets for therapeutic intervention and biomarkers for prediction of tumour metastasis. Canine Gene 1.1 ST Arrays were employed for genome-wide expression analysis of formalin-fixed, paraffin-embedded biopsies of mast cell tumours borne by dogs that either died due to confirmed mast cell tumour metastasis, or were still alive more than 1000 days post-surgery. Decreased gene expression in the metastasising tumours appears to be associated with a loss of cell polarity, reduced cell-cell and cell-ECM adhesion, and increased cell deformability and motility. Dysregulated gene expression may also promote extracellular matrix and base membrane degradation, suppression of cell cycle arrest and apoptosis, and angiogenesis. Down-regulation of gene expression in the metastasising tumours may be achieved at least in part by small nucleolar RNA-derived RNA and microRNA-effected gene silencing. Employing cross-validation, a linear discriminant analysis-based classifier featuring 19 genes that displayed two-fold differences in expression between metastasising and non-metastasising tumours was estimated to classify metastasising and non-metastasising tumours with accuracies of 90–100% and 70–100%, respectively. The differential expression of 9 of the discriminator genes was confirmed by quantitative reverse transcription-PCR.

## Introduction

Canine mast cell tumours (MCTs) are neoplastic proliferations which predominantly arise from tissue mast cells in the dermal layer of the skin [1]. MCTs are the most common canine skin tumour [2] with an estimated incidence of 129 per 100,000 dogs. Although most breeds are affected, several breed predispositions have been reported [3]. The majority of MCTs are successfully treated by surgery and/or radiotherapy, but approximately 25% of tumours spread to a regional lymph node, spleen and/or liver, and with local therapy alone death usually follows within 1 year of diagnosis. Dogs that have metastatic disease, or are believed to have a high risk of developing metastatic disease, are often treated with adjunctive chemotherapy. An overall response rate of 47%, with a median response duration of 154 days, has been reported for treatment of measureable MCTs with an often used vinblastine and prednisolone protocol [4], and a median survival time of 1374 days was achieved when an equivalent protocol was utilised post-surgery [5]. However, the side-effects of chemotherapy may include myelosuppression, neutropenia and gastrointestinal disorders.

MCTs are usually classified by histological grade, which is the most important single prognostic factor [1]. Currently, two grading systems are used. The Patnaik grading system is well established and assigns a MCT to one of 3 grades (I, II and III) according to descriptive histological criteria [6]. However, Patnaik grade does not predict metastasis; <10% of grade I, 5–22% of grade II, and >80% of grade III MCTs metastasise [7]. The more recent binary Kiupel histologic grading system [8] utilises more numerical and fewer descriptive criteria and was devised to reduce the discord observed between pathologists applying the Patnaik system. Whilst Kiupel grade is associated with survival time, it also cannot accurately predict metastasis; 37.5% of tumours classified as ‘low grade’ were borne by dogs with distant metastases, whilst 21.9% of MCTs designated as ‘high grade’ were from dogs without distant metastases [9].

Decreased MCT patient survival has been associated with ‘high’ mitotic index [10], increases in the number of Ki-67 positive nuclei [11], argyrophylic nucleolar organizer regions [11] and minichromosome maintenance protein 7 positive cells [12], respectively, and decreased expression of the cell adhesion molecule TSLC1 [13] in cutaneous MCTs. However, although significant differences between the proliferating cell nuclear antigen and argyrophilic nucleolar organizing region counts for metastasising and non-metastasising MCTs were described in a single study [14], neither cell proliferation index has subsequently been shown to be capable of predicting canine cutaneous MCT metastasis [15].

Metastasis is a complex process, each step of which is thought to rely at least in part on cells acquiring specific genetic and/or epigenetic alterations [16] additional to those that drive primary tumour development. The alterations may effect changes in gene expression, and metastasis-associated gene expression signatures have been identified for a number of human tumours [17]. Metastasis-associated gene expression signatures may predict metastasis [18], and several form the basis of routine prognostic tests; e.g. the DecisionDx-UM test [19]. Differential gene expression analysis has also identified biological processes involved in metastasis [20] and candidate metastasis-suppressor [21] and promoting genes [22]. Gene expression analysis of canine cutaneous MCTs was recently performed [23] with the intention of evaluating whether biological behaviour could be predicted on the basis of tumour gene expression profile. However, differential expression analysis compared ‘differentiated’ and ‘undifferentiated’ tumours designated solely on the basis of Kiupel grade [8], with no reference to the presence or absence (or later development) of metastasis.

Clinical management of canine cutaneous MCT would be greatly assisted by the capability to predict tumour metastasis, whilst targeted prevention of metastasis would ultimately represent the most effective life-saving strategy. Elucidation of the molecular genetic contributions to canine cutaneous MCT metastatic progression affords a means of identifying biomarkers of metastasis and potential targets for therapeutic intervention.

In the current study we compared the global gene expression profiles of formalin-fixed, paraffin-embedded biopsies (FFPE) of primary cutaneous MCTs that did and did not metastasise. The aim was to identify genes that are associated with the metastatic progression of cutaneous MCTs, and evaluate the potential for differentiating metastasising and non-metastasising MCTs on the basis of a metastasis-associated gene expression signature.

## Materials and methods

### Ethics statements

This study was approved by the Animal Health Trust and the University of Liverpool ethics committees, respectively. Informed, written consent was obtained from the owner of each dog whose MCT biopsy was included in this study. A MCT biopsy could be withdrawn from the study at any time. Patient treatment was unaffected by the study.

### Tumour samples

Diagnostic histopathology FFPE biopsies of canine primary cutaneous MCTs were collected from dogs treated in the Clinical Oncology departments at the Animal Health Trust Centre for Small Animal Studies and the University of Liverpool Small Animal Teaching Hospital, respectively, between 1997 and 2010. The biopsies were from dogs that were treated for a solitary cutaneous MCT, and for which complete staging information (at the time of initial presentation to the referral hospital), and follow-up information to the time of patient death or a minimum of 1000 days following diagnosis (whichever came first) were available. The occurrence of metastasis was determined by abdominal ultrasound or computed tomography, and cytological/histological examination of a biopsy of one or more regional/draining lymph nodes. For a cytological diagnosis of lymph node metastasis, mast cells had to appear in clusters or sheets, or appear grossly abnormal [14]. MCT biopsies were designated as ‘metastasising’ (M) if they were borne by dogs which died or were euthanased due to MCT metastatic disease <560 days post-surgery/biopsy (regardless of adjuvant chemotherapy, including prednisolone, and/or radiotherapy), *and* for whom metastasis was confirmed by diagnostic imaging *and* pathological analysis. Non-metastasising (NM) MCT biopsies were removed from dogs which received no adjuvant therapy (including prednisolone) *and* were still alive >1000 days post-surgery/biopsy [24], *and* for whom metastasis was not identified by imaging or pathological analysis.

### RNA isolation and purification

Total RNA was isolated from FFPE MCT biopsies using the RecoverAll Total Nucleic Acid Isolation Kit, which incorporates on-column DNase digestion (ThermoFisher Scientific, Paisley, UK). RNA was treated with Heparinase I (Sigma, Gillingham, UK) (10U/μg RNA) in 5mM Tris-HCl (pH7.5), 1mM CaCl_2_, 4U/μl RNasin Plus RNase Inhibitor (Promega, Southampton, UK) for 3h at 25°C, and subject to further DNase digestion (TURBO DNA-free kit; ThermoFisher Scientific, Paisley, UK). RNA was purified (RNA Clean & Concentrator-5; Zymo Research, Freiburg, Germany) and quantified by RiboGreen fluorometry (Quant-iT RiboGreen RNA Assay Kit, ThermoFisher Scientific, Paisley, UK).

### RNA sample selection

The integrity of each FFPE MCT RNA sample was assessed by reverse transcription-quantitative PCR (RT-qPCR) assay of the copy number of a 126bp fragment of a 130–150bp short interspersed nuclear element (SINE) present every 5–8.3kb in the canine genome [25], and shown (by BLAST similarity search against canine mRNA sequences) to occur in the 3’-untranslated region of hundreds of canine mRNAs. cDNA was prepared from 10ng of each total RNA sample using the High-Capacity cDNA Reverse Transcription Kit (ThermoFisher Scientific, Paisley, UK), and triplicate PCR assays were performed (PowerUp SYBR Green Master Mix; ThermoFisher Scientific, Paisley, UK) using 1μl aliquots of a 1 in 1.6-fold dilution of each cDNA sample. A quantification cycle (Cq) value was derived for each PCR product using the PCR machine software (StepOne Plus; ThermoFisher Scientific, Paisley, UK), and a geometric mean Cq value calculated for each MCT cDNA sample as a measure of RNA integrity.

Additional details are included in S1 File and S1 Table.

### Global gene expression profiling

#### RNA amplification, labelling and microarray hybridisation

Fragmented, biotinylated double-stranded cDNA was prepared from 50ng of each FFPE MCT RNA sample using the SensationPlus FFPE amplification and WT labelling Kit (ThermoFisher Scientific, Paisley, UK), and hybridised (in groups of 4) to a Canine Gene 1.1 ST Array Strip (ThermoFisher Scientific, Paisley, UK). Post-hybridisation washing and staining, and array scanning were performed using the GeneAtlas System Fluidics and Imaging Stations (ThermoFisher Scientific, Paisley, UK), respectively.

#### Microarray data analysis

Exon-level probe set expression values were generated by quantile normalisation, log_2_ transformation and signal summarisation, performed using the Robust Multichip Analysis algorithm, implemented within ‘Affymetrix Expression Console Software 1.3’ (ThermoFisher Scientific, Paisley, UK). ‘Outlier arrays’ were considered to be those that had any single sample quality, labelling quality and hybridisation quality metric value ≥2 standard deviations away from the mean of the metric value for all the arrays [26]. Outlier arrays were excluded, and processing of the raw probe-level signal intensity data repeated to generate both quantile normalised and log_2_-transformed exon and gene-level probe set expression values. Gene-level probe sets (‘Transcript clusters’) with ‘crosshyb_type’ = 1 (unique hybridisation target) and ‘category’ = ‘main’ annotations, and for which at least 1 exon probe set was ‘present’ (detection above background p-value <0.01; [27]) in at least 30% of the tumours in the NM and/or M MCT cohort, were considered to be expressed in the MCTs and were used for subsequent analyses.

Relationships between MCT gene-level expression profiles were visualised by hierarchical clustering (average linkage; similarity metric = Pearson Correlation Coefficient) performed using Cluster [28]. Genes displaying statistically significant differences in expression between M and NM MCTs were identified using a two-tailed t-test for unpaired data. P-values were adjusted by permutation testing [29]. The potential identities of differentially expressed Transcript clusters that represented ‘predicted genes’, or for which no annotation was available, were sought by BLAST similarity search of Transcript cluster sequences against canine and human mRNAs and non-coding RNAs, respectively.

#### Functional annotation analysis

Over represented functional annotations associated with the differentially expressed genes were identified using DAVID [30] by comparison with the functional annotations attributed to all the ‘crosshyb_type’ = 1 and ‘category’ = ‘main’ annotated Transcript clusters for which at least 1 exon probe set was ‘present’ in at least 30% of the tumours in the NM and/or M MCT cohort.

### Reverse transcription-quantitative PCR (RT-qPCR)

The differential expression of selected genes was validated by RT-qPCR. A TaqMan or SYBR Green PCR assay was designed (Beacon Designer, Premier Biosoft; Palo Alto, USA) for each Transcript cluster based upon a unique region within the sequence of the constituent exon probe set that showed the largest statistically significant difference in expression between the M and NM MCTs. Prior to use in RT-qPCR, each MCT cDNA was assayed for the presence of PCR inhibitors (S1 File and S2 Table). Triplicate PCR assays for each preamplified MCT cDNA sample were run on an ABI StepOne Plus PCR machine (ThermoFisher Scientific, Paisley, UK), and a geometric mean Cq value derived. For use as a ‘reference gene’ for normalisation of target gene expression measurements [31], the copy number of a 71bp fragment of a SINE [25] that occurs in the 3’-untranslated region of hundreds of canine mRNAs in each MCT RNA sample was also assayed. MCT Cq values with a standard deviation >0.5 were excluded from further analyses, and genes with a geometric mean C_q_ of ≥35 were considered not to be expressed. Additional details are included in S1 File and S1 Table.

#### RT-qPCR data analysis

The geometric mean C_q_ measures of target gene expression were imported into qbase+ (Biogazelle, Gent, Belgium) and each converted to a relative measure of gene expression (‘Normalised Relative Quantity; NRQ [32]) using a normalisation factor derived from the respective geometric mean canine SINE [25, 31] C_q_ value. The statistical significance of differences in the expression of genes between M and NM MCTs was determined using a two-tailed t-test for unpaired data performed on log_10_ transformations of the NRQs.

### Class prediction analysis

The optimal classification function for gene expression data-based prediction of ‘metastatic status’ (M or NM) was identified by evaluation of the characteristics of the expression values obtained for the Transcript clusters expressed in the MCTs using the R package SPreFuGED [33], which predicts the performance of representatives of 10 classification functions. Class prediction by Linear Discriminant Analysis was performed using the *lda* function provided by the R Package MASS [34]. The accuracy of class prediction was estimated through testing by cross-validation. The expression profiles (selected genes) of the MCTs were randomly partitioned into a ‘training data set’ (comprising data for ~90% of the MCTs) and a ‘test data set’ (comprising data for two M and one NM MCT), and the class (M or NM) of the MCTs comprising the test data set predicted. Ten training and test data set combinations were evaluated. The *lda* function was also run in the ‘leave-one-out cross-validation mode’, whereby the class of each MCT was predicted whilst using the expression data for the remaining (n-1) MCTs as a training data set.

## Results

### Tumours selected for gene expression profiling

MCT biopsies from 78 dogs were eligible for inclusion in the study. PowerAtlas [35] analysis of Gene Expression Omnibus [36] datasets derived for several human tumours estimated that using 20 tumour samples in each of two ‘outcome groups’ would afford an ‘Estimated Discovery Rate’ (Power) of 73.1–81.7% at the 0.05 significance level. Consequently, the integrity of each MCT RNA was assessed to enable compilation of ‘M’ and ‘NM’ MCT sample groups comprising 20 RNA samples with a similar range of integrities (S3 Table).

### Tumours included in differential expression analysis

Sample quality metrics associated with exon-level probe set expression profiles of 40 MCTs were reviewed to identify tumours whose expression profiles differed significantly from the majority of the cohort. Array data for 2 M MCTs and 4 NM MCTs were excluded (S3 and S4 Tables) because for each the ‘percent of probe sets detected above background’ differed by >2 standard deviations from the cohort mean value [26]. Gene-level probe set expression data for 18 M MCTs and 16 NM MCTs was re-processed for further analysis featuring 5,207 Transcript clusters annotated as ‘crosshyb_type’ = ‘1’ and ‘category’ = ‘main’ probe sets, and for which at least 1 exon probe set was ‘present’ in at least 30% of the tumours in the NM and/or M MCT cohort. The raw and processed microarray data has been deposited in the NCBI Gene Expression Omnibus repository (GEO series accession number GSE122590).

The characteristics of the dogs that bore the 18 M MCTs and 16 NM MCTs tumours are detailed in Tables 1 and 2. Nine breeds were represented in the M group and 7 in the NM group, with 5 breeds common to both groups. Multiple representatives of a single breed probably reflect both breed popularity and an increased susceptibility to MCT development [3]; for example, MCTs borne by Labrador Retrievers represent 19% of the NM MCTs and 39% of the M MCTs. Equal proportions of both sexes were represented in the M MCT biopsy group, whilst 69% of the NM MCTs were borne by male dogs. Interestingly, the median age of the dogs affected by M MCTs was 3 years higher than that of the dogs that developed NM tumours. Unsupervised hierarchical clustering of the 34 MCTs on the basis of the expression values of the 20% of Transcript Clusters (1,041) with the highest variance in expression signal, gave no indication of an association between global MCT gene expression profile and breed, sex, or age at diagnosis, respectively (S1 Fig). The mean age of a FFPE NM MCT specimen was 1.6 x higher than that of a FFPE M MCT biopsy, although the FFPE tumour biopsy age does not correlate with tumour RNA integrity (Spearman rank correlation coefficient = 0.13; S3 Table).

**Table 1:** Dogs bearing metastasising cutaneous mast cell tumours included in differential gene expression analysis.

Dog ID.	Breed	Sex^a^	Age at diagnosis (Years)
**B1**	Boxer	Fe	9
**CB6**	Cross breed	FeN	9
**CB4**	Cross breed	FeN	10
**CB1**	Cross breed	FeN	15
**CCR1**	Curly Coated Retriever	Fe	6
**D1**	Dogue de Bordeaux	MaN	2
**GS1**	German Shepherd	Ma	9
**GR1**	Golden Retriever	FeN	8
**HV1**	Hungarian Vizsla	MaN	7
**LR10**	Labrador Retriever	FeN	4
**LR1**	Labrador Retriever	FeN	8
**LR2**	Labrador Retriever	Ma	7
**LR5**	Labrador Retriever	Ma	8
**LR9**	Labrador Retriever	Ma	10
**LR8**	Labrador Retriever	MaN	6
**LR3**	Labrador Retriever	MaN	8
**SBT1**	Staffordshire Bull Terrier	FeN	10
**W1**	Whippet	MaN	11
		Mean and standard deviation	8.17 ± 2.73
		Median	8.00
		Interquartile range	3.25

**Table 2:** Dogs bearing non-metastasising cutaneous mast cell tumours included in differential gene expression analysis.

Dog ID.	Breed	Sex^a^	Age at diagnosis (Years)
**B4**	Boxer	Fe	5
**B2**	Boxer	FeN	4
**B3**	Boxer	Ma	4
**CB3**	Cross breed	FeN	10
**CB2**	Cross breed	FeN	11
**CB5**	Cross breed	MaN	5
**ETT1**	English Toy Terrier	Ma	6
**GR2**	Golden Retriever	Ma	3
**GR4**	Golden Retriever	Ma	7
**GR3**	Golden Retriever	MaN	5
**LR4**	Labrador Retriever	Ma	6
**LR6**	Labrador Retriever	MaN	3
**LR7**	Labrador Retriever	MaN	5
**MS1**	Miniature Schnauzer	MaN	3
**SBT2**	Staffordshire Bull Terrier	MaN	6
**W2**	Whippet	Fe	5
		Mean and standard deviation	5.50 ± 2.21
		Median	5.00
		Interquartile range	2.00

^a^Fe: Female; FeN: Neutered female; Ma: Male; MaN: Neutered male

### Genes differentially expressed between M and NM MCTs

**Fig 1:**
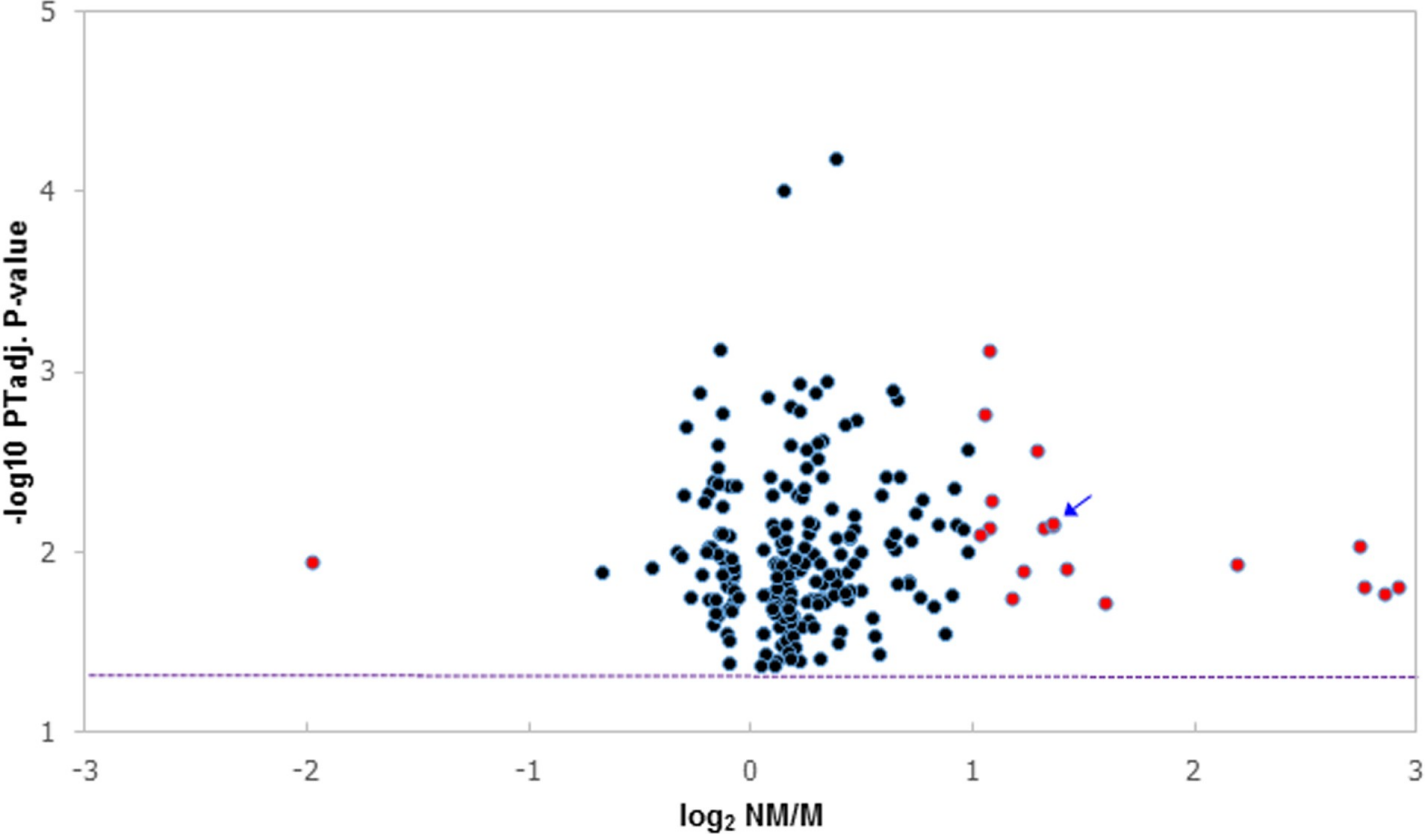
Genes exhibiting differential expression between metastasising and non-metastasising MCTs. Expression of 218 genes in 18 metastasising (M) and 16 non-metastasising (NM) MCTs. Each sphere represents an individual gene. The difference in expression between the M and NM MCTs is represented (x-axis) by the log_2_-transformed fold-change (NM/M). Red spheres denote the 19 genes which exhibit a difference in expression of ≥2.0 (either NM>M, or M>NM). The arrow indicates two genes which cannot be resolved by their x, y co-ordinates. The statistical significance of differences in expression between NM and M MCTs is denoted (y axis) by the minus log_10_-transformed permutation testing-adjusted unpaired t-test derived p-values. A -log10 PTadj. p-value equivalent to a PTadj. p-value = 0.05 is indicated by the dotted line.

**Fig 2:**
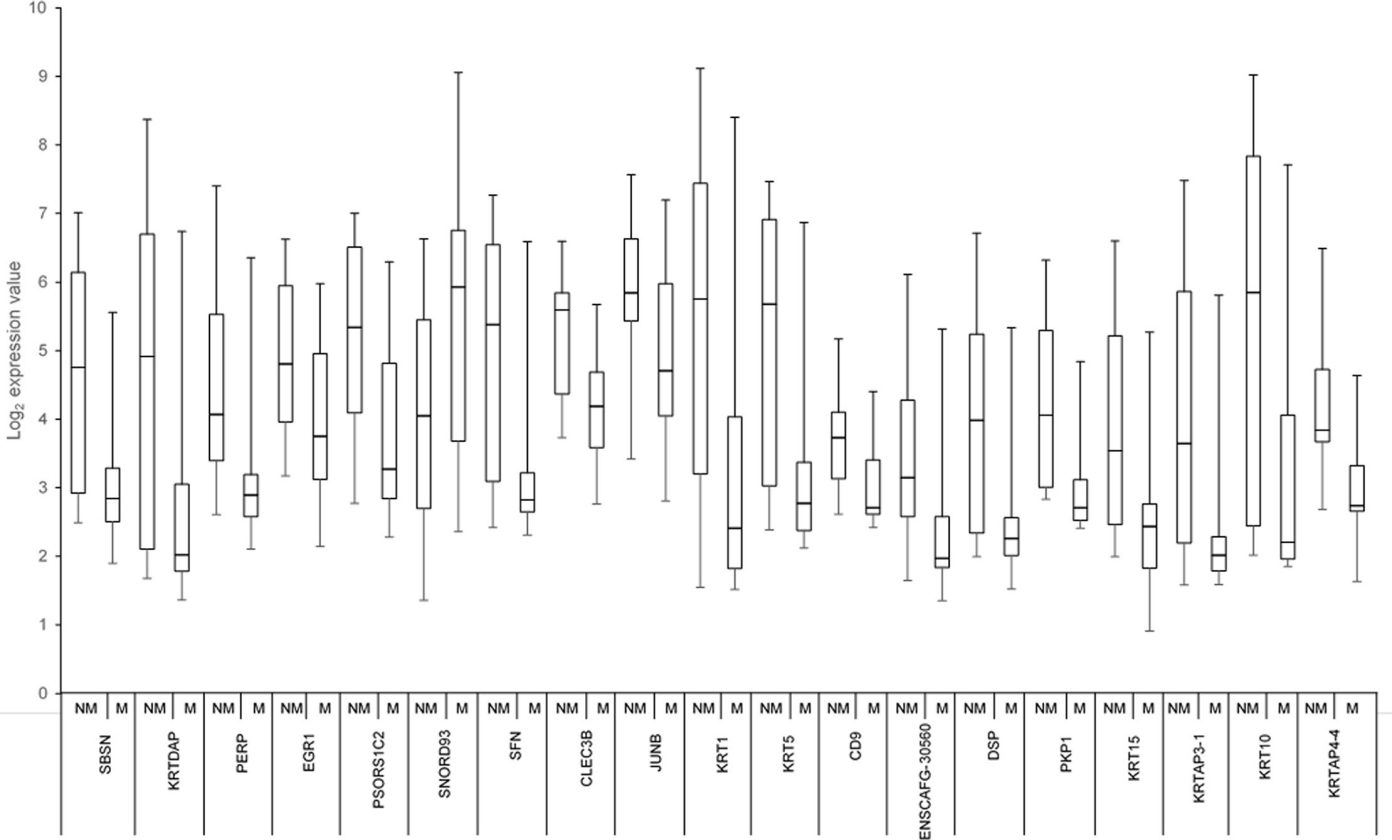
Expression levels of 19 genes that display >two-fold differences in expression between metastasising and non-metastasising MCTs. The bottom and top of each box denote the expression measurements that encompass the values shared by 25% and 75% of the tumours, respectively. The line within each box represents the median expression value, whilst the vertical lines extending above and below each box indicate the maximum and minimum expression values, respectively. M = metastasising tumour; NM = Non-metastasising MCT.

A statistically significant difference in expression (permutation-testing adjusted p-value <0.05) between M and NM MCTs was observed for 218 Transcript clusters; 162 displayed increased expression in the NM MCTs and 56 were expressed at a higher level in the M MCTs (Fig 1). Nineteen genes displayed a >two-fold difference in expression between the M and NM MCTs; 18 of the genes showed increased expression in the NM MCTs (Table 3; Fig 2). Gene annotation was not available for 2 of the Transcript clusters, although each displayed significant sequence similarity to one, or more, mRNAs encoded by a single canine gene. Three genes that exhibited a >two-fold difference in expression are located on each of chromosome 1, 9, and 27, respectively. Two of the genes (SBSN and KRTDAP) are positioned adjacent to each other in a 37.6kb region on chromosome 1 (Table 3). Two chromosomes (CFA14 and CFA31) harbour a higher proportion of the 218 differentially expressed genes than may be expected if their chromosomal distribution only reflected the chromosomal assignments of the genes expressed in the MCTs (S5 Table). Three differentially expressed keratin-associated genes (KRTAP8-1, KRTAP7-1 and KRTAP11-1), which display decreased expression in the M MCTs, lie adjacent to each other within a 69.7kb region on CFA31 (S5 Table).

**Table 3:** Genes displaying >two-fold differences in expression between metastasising and non-metastasising MCTs.

Gene description (Gene symbol/ID.)	Chromosomal location^b^^[^^37^^]^	Fold change (NM/M)^d^	Adj_p-value^e^
**TP53 apoptosis effector** (*PERP*)	1: 30.42	2.11	0.008
**Suprabasin** (*SBSN*)	1: 117.13^c^	2.84	0.012
**Keratinocyte differentiation-associated protein** (*KRTDAP*)	1: 117.17^c^	7.23	0.017
**Stratifin** (*SFN*)	2: 73.27	4.71	0.012
**Plakophilin 1** (*PKP1*)	7: 1.92	2.45	0.003
**Keratin 15** (*KRT15*)	9: 21.25	2.12	0.005
**Sequence similarity to Keratin-associated protein 4–4 (E-val: 0.0; 470bp; 26%)** (*KRTAP4-4*)^a^	9: 21.55	2.10	0.001
**Keratin-associated protein 3–1** (*KRTAP3-1*)	9: 21.68	2.60	0.006
**Keratin 10** (*KRT10*)	9: 21.86	8.50	0.014
**Early growth response 1** (*EGR1*)	11: 26.05	2.53	0.009
**Psoriasis susceptibility 1 candidate 2** (*PSORS1C2*)	12: 0.84	3.17	0.019
**Small nucleolar RNA, C/D box 93** (*SNORD93*)	14: 36.56	0.25	0.011
**C-type lectin domain family 3, member B** (*CLEC3B*)	20: 43.32	2.57	0.007
**junB proto-oncogene** (*JUNB*)	20: 49.36	2.25	0.018
**Epithelial keratin 1** (*KRT1*)	27: 2.42	7.28	0.010
**Keratin 5** (*KRT5*)	27: 2.57	6.81	0.013
**CD9 antigen** (*CD9*)	27: 38.74	2.07	0.002
**Sequence similarity to ENSCAFG00000030560 (E-val: 4 x 10**^**−54**^**; 106bp; 60%)** (ENSCAFG-30560)^a^	31: 38.47	2.05	0.007
**Desmoplakin** (*DSP*)	35: 7.48	2.48	0.013

^a^Transcript cluster with no gene annotation. The most significant similarity between the sequence (spliced exons) of the Transcript cluster and a canine mRNA is listed. The significance of the sequence similarity is denoted by the E value and the length of the sequence alignment, and the proportion of the Transcript cluster sequence included in the alignment is stated.

^b^Chromosomal location is denoted by the chromosome name and the gene start base co-ordinate.

^c^The genes encoding suprabasin and keratinocyte differentiation-associated protein are adjacent to each on chromosome 1.

^d^Ratio of median gene-level expression values.

^e^Permutation testing-adjusted t-test p-value

### Functional annotation enrichment analysis

In order to identify biological processes and pathways involved in MCT metastasis, functional annotations over-represented amongst those assigned to the 218 Transcript clusters differentially expressed between the M and NM MCTs were identified by comparison with those attributed to the 5,207 Transcript clusters for which at least 1 exon probe set was ‘present’ in at least 30% of the tumours in the NM and/or M MCT cohort. The frequencies of functional annotations available for 177 of 209 differentially expressed Transcript clusters for which an Ensembl Gene ID [37] could be defined were compared with those available for 4,846 of the ‘present’ Transcript clusters which had an Ensembl Gene ID. Six Gene Ontology Consortium biological processes and two KEGG pathways were enriched amongst the differentially expressed genes (Table 4).

**Table 4:** Differentially expressed gene-associated enriched functional annotations.

			Gene expression	
Functional annotation^a^	Fold enrichment^b^	P-value^c^	NM > M	M > NM
**GO BP: 0016337 single organismal cell-cell adhesion**	6.699	0.005	*CDSN*, *DSP*, *PKP1*, *SCRIB*	
**GO BP: 0008285 negative regulation of cell proliferation**	3.240	0.006	*CD9*, *ETV3*, *HDAC4*, *NF2*, *SFRP4*, *SPRY1*, *STRN*, *TFAP2A*	*DNAJA3*
**GO BP: 0060070 canonical Wnt signaling pathway**	6.029	0.008	*BCL9L*, *MYC*, *PLPP3*, *SDC1*, *SFRP4*	
**GO BP: 0051496 positive regulation of stress fiber assembly**	7.420	0.014	*ARHGEF10*, *BRAF*, *EVL*, *NF2*	
**GO BP: 0008219 cell death**	12.058	0.023	*AXIN1*, *EMP2*, *PMP22*	
**GO BP: 0032060 bleb assembly**	10.336	0.031	*EMP1*, *EMP2*, *PMP22*	
**KP cfa04024: cAMP signaling pathway**	2.969	0.045	*AFDN*, *BRAF*, *CREBBP*, *FOS*	*CALM3*
**KP cfa05210: Colorectal cancer**	4.783	0.046	*BRAF*, *FOS*, *MYC*, *PIK3R2*	

^a^GO BP: Gene Ontology Biological Process; KP: Kegg Pathway

^b^Fold enrichment—Proportion of 177 differentially expressed genes with the functional annotation/proportion of ~4,846 genes expressed in the MCTs that have the functional annotation.

^c^P-value: Fisher Exact test p-value (EASE score) modified to reduce false positive results.

### Validation of differential expression by RT-qPCR

The expression levels of 9 of the genes which showed >two-fold differences in expression between 18 M and 16 NM MCTs were measured by RT-qPCR assay of the same MCT biopsies (Table 5). The genes selected for validation of differential expression included the only gene that displayed >two-fold increased expression in the M MCTs, the 2 genes represented by Transcript clusters for which gene annotation was not available, and genes representative of different enriched functional annotations (Table 4) and/or associated with different biological processes. Valid gene expression measurements (Cq values) were obtained for fewer than the 34 MCT biopsies assayed because either the Cq was ≥35 or was ‘undetermined’, or the Cq standard deviation for triplicate assays was >0.5. There was a high degree of concordance between the expression levels calculated for individual MCTs (indicated by Spearman rank correlation coefficients in Table 5), and between NM/M fold changes, measured by microarray (exon-level probe set) and RT-qPCR, respectively. For 2 of the genes (*EGR1* and *KRT10*), the differences in expression between the M and NM MCTs was statistically significant. However, statistical significance is affected by both the number of samples and which samples are included in a statistical test. The differences in expression between the NM and M groups attained statistical significance for *KRT10* and *PERP* when the microarray-derived expression measurements for only the MCTs that were included in the statistical analysis of the RT-qPCR generated expression data were analysed (Table 5).

**Table 5:** Differences in gene expression between M and NM MCTs measured by RT-qPCR.

				RT-qPCR		
Gene symbol/ID.	Exon-level fold change^a^ (NM/M)	No. NM MCTs^b^	No. M MCTs^b^	Fold change^c^ (NM/M)	Spearman RCC^d^	p-value^e^ (Array)
***CD9***	2.25 (1.80)	12	8	2.78	0.51	0.405 (0.090)
***DSP***	19.08 (1.48)	7	4	40.43	0.74	0.217 (0.152)
***EGR1***	3.66 (2.11)	11	14	6.05	0.69	0.028 (0.050)
**ENSCAFG-30560**	2.05 (1.43)	5	8	7.15	0.81	0.139 (0.213)
***KRT10***	23.43 (43.37)	11	12	53.87	0.88	0.009 (0.017)
***KRTAP4-4***	2.10 (2.77)	7	7	1.12	0.76	0.730 (0.208)
***PERP***	7.38 (4.69)	11	10	9.94	0.80	0.083 (0.041)
***SBSN***	3.49 (2.62)	10	6	14.77	0.81	0.140 (0.115)
***SNORD93***	0.25 (0.39)	12	16	0.37	0.50	0.196 (0.258)

^a^Fold change differences in expression between 18 M and 16 NM MCTs determined by microarray—Ratio of median expression values for the Exon probe set upon which RT-qPCR assay design was based. In parenthesis are the fold change differences calculated when only the microarray-derived expression values of MCTs that yielded valid Cq values in RT-qPCR assay were considered.

^b^The numbers of NM and M MCTs represent the numbers of samples for which valid Cq (Cq <35; Cq SD<0.5) measurements were obtained. ‘Non-valid’ Cq values were attributable to: Cq <35 or ‘undetermined’ and Cq SD>0.5.

^c^Fold change determined by RT-qPCR assay.

^d^The Spearman rank correlation coefficient (RCC) indicates the extent of the concordance between the expression values for individual MCT assayed by microarray and RT-qPCR, respectively.

^e^The statistical significance of the RT-qPCR measured differences in expression between the NM and M MCTs determined by t-test. The statistical significance of the differences between the microarray-derived gene-level expression values measured for the same MCTs is shown in parenthesis.

### Class prediction analysis

**Fig 3:**
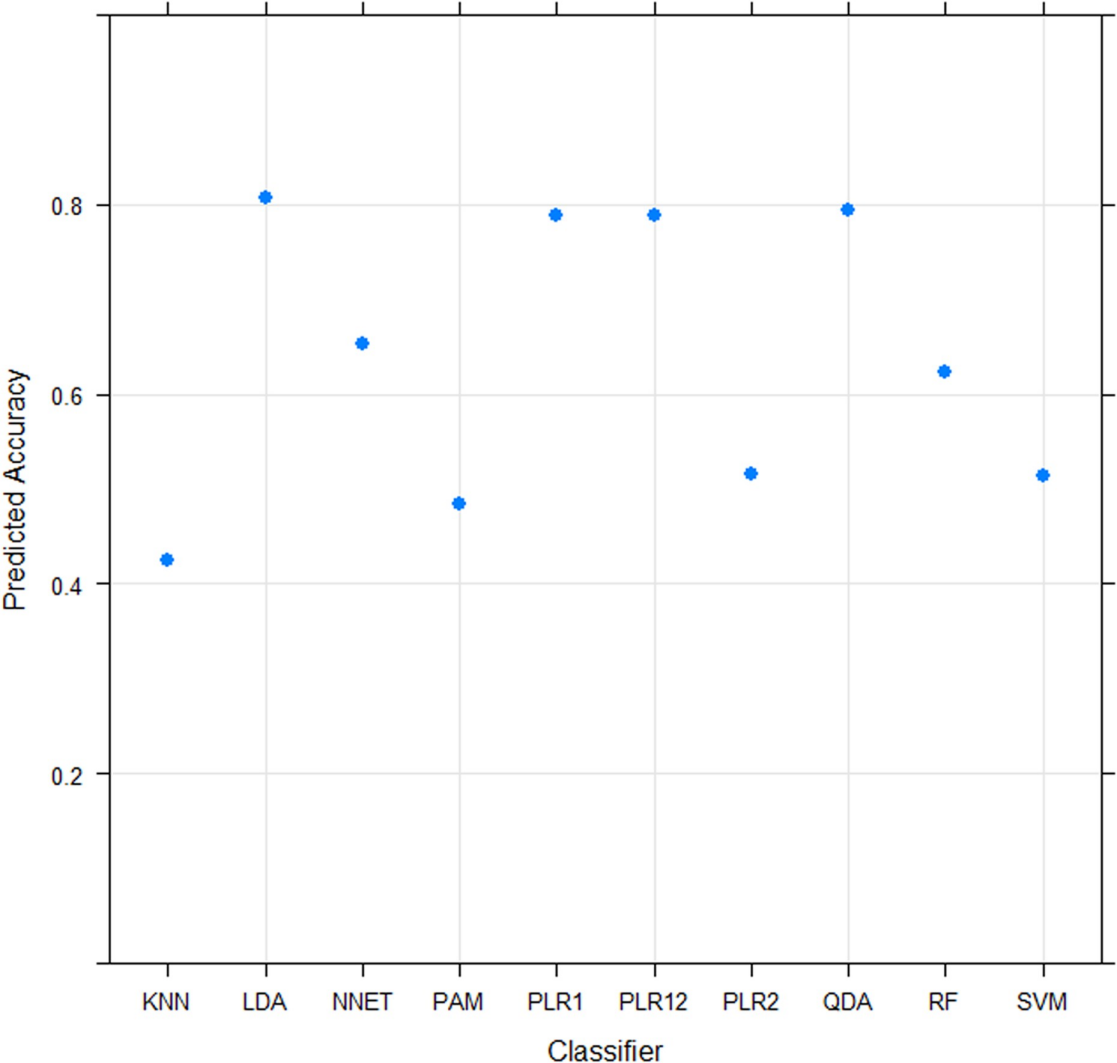
Predicted accuracy of classification functions on the basis of the characteristics of the expression values obtained for 5,207 Transcript clusters ‘present’ in the MCTs. Classification functions evaluated by SPreFuGED [33]: KNN—k-nearest neighbours, LDA—linear discriminant analysis, NNET—feed-forward neural network, PAM—prediction analysis of microarrays, PLR1 - ℓ1ℓ1 penalized logistic regression (Lasso), PLR12 - ℓ1ℓ1 and ℓ2ℓ2 penalized logistic regression (Elastic net), PLR2 - ℓ2ℓ2 penalized logistic regression (Ridge), QDA—quadratic discriminant analysis, RF—Random forest, SVM—support vector machine.

**Fig 4:**
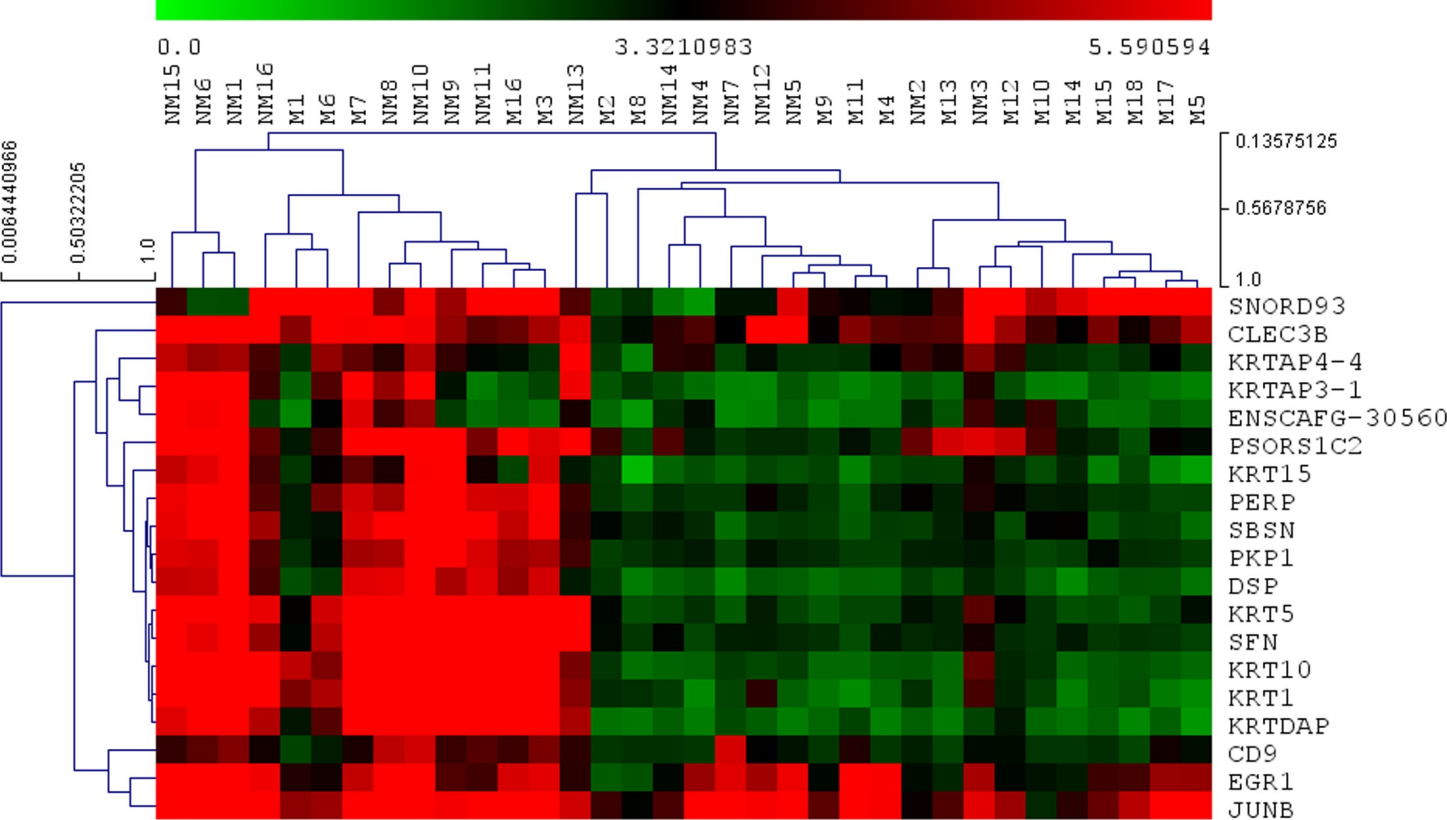
Hierarchical clustering of 18 M and 16 NM MCTs, and 19 genes which display >two-fold differences in expression between M and NM MCTs. The horizontal colour bar denotes the log_2_ expression value. The extent of the dissimilarity (equal to 1 minus the Pearson correlation coefficient) between MCTs is indicated by the vertical scale bar in the top right hand corner of the figure, and between genes is indicated by the horizontal scale bar in the top left hand corner of the figure.

**Fig 5:**
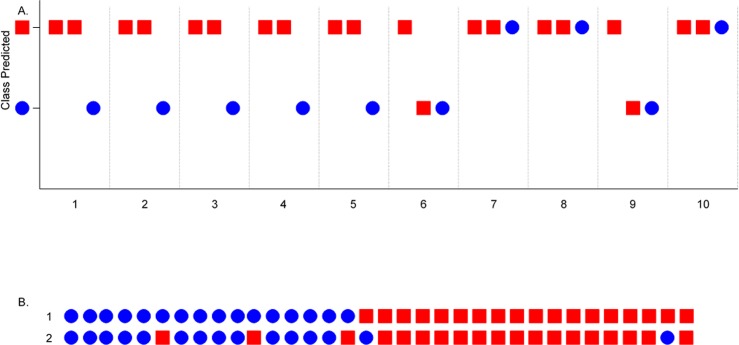
Class prediction by Linear Discriminant Analysis. (A) Results of predicting the classes (M = a square, and NM = a circle) of 3 MCTs (2 M MCTs and 1 NM MCT) on the basis of the expression values of the 19 genes which show statistically significant >two-fold differences in expression between M and NM MCTs. Class prediction was performed on 10 occasions. On each occasion the gene expression data for 34 MCTs (18 M and 16 NM) was randomly divided into a ‘training set’ (90% of the data, comprising 16 M and 15 NM MCTs) and a ‘test set’ (10% of the data, representing 2 M MCTs and 1 NM MCT). The LDA classifier was trained using the training data set and then used to assign the 3 ‘test MCTs’ to either the M or NM class. (B) Results of predicting the class of each of 34 MCTs (16 NM = circles, 18 M = squares) on the basis of the expression values of the 19 genes which show statistically significant >two-fold differences in expression between M and NM MCTs. Over 34 iterations the LDA classifier was trained using the expression data for 33 of the 34 MCTs, and the classification of the remaining MCT predicted. The actual class of each MCT is depicted in row 1 and the predicted class of each MCT is shown in row 2.

Linear Discriminant Analysis (LDA) was predicted to be the optimal classification function for prediction of ‘metastatic status’ (M or NM) on the basis of the characteristics of the expression values obtained for the 5,207 Transcript clusters ‘present’ in the MCTs (Fig 3). Hierarchical clustering of the 18 M and 16 NM MCTs, on the basis of the microarray-measured expression levels of the 19 genes which displayed >two-fold differences in expression between the M and NM MCTs, separated the MCTs into 2 major groups (Fig 4). The largest group contained 72% of the M MCTs, whilst the NM MCTs were equally divided between the two groups. Although the M and NM MCTs were not partitioned into two groups, class prediction by LDA does not assign a sample to a class on the same basis (i.e. using a measure of the ‘distance’ between 2 samples/pre-created sample groups equal to 1 minus the correlation coefficient) that samples are agglomeratively grouped by hierarchical clustering. Consequently, and as the fold difference in the expression of a gene between classes has been shown to be an effective criterion for ranking genes for use in class prediction [38, 39], the efficacy of using the 19 genes which displayed >two-fold differences in expression between M and NM MCTs in class prediction was evaluated. The performance of the LDA classifier was tested by cross-validation, measuring the accuracy with which 2 M MCTs and 1 NM MCT (randomly selected) were classified (as M or NM) on each of 10 occasions (after the classifier had been trained using the expression values for the remaining 16 M and 15 NM MCTs) (Fig 5). Mean classification accuracies of 90% (M MCTs) and 70% (NM MCTs) were estimated, whilst a median classification accuracy of 100% was achieved for both M and NM MCTs. Evaluating the performance of the classifier in a ‘leave-one-out cross-validation mode’, 88.9% of the 18 M MCTs were correctly classified and 81.3% of the 16 NM MCTs were correctly assigned to the NM class.

## Discussion

Mast cell tumours are one of the most common tumours affecting dogs. There is currently no accurate way of predicting whether a tumour is one of the 20–30% of MCTs that will metastasise, and pre-existing micrometastases may not be detected by current imaging modalities. Activating internal tandem duplications (ITDs) in exon 11 of *c-kit* have been reported in 9% of canine MCTs [40]. The mutations affect the juxtamembrane domain and are associated with a higher histological grade and poor prognosis [40], although they are present in less than 50% of ‘high grade’ MCTs [40, 41]. In the absence of an effective predictive test for MCT metastasis, dogs that bear tumours with unrecognised metastatic potential may not receive adjuvant chemotherapy, whilst dogs erroneously believed to harbour a metastasising tumour may be unnecessarily exposed to the possible side-effects of chemotherapy. The molecular genetic drivers of canine MCT metastasis potentially represent both biomarkers of metastasis and targets for anti-metastasis therapeutics. In the current study we sought to identify ‘across breed’ molecular genetic contributants to canine MCT metastasis by comparing global gene expression in 20 MCTs that metastasised with that in 20 MCTs that did not metastasise. The tumours profiled were borne by 11 breeds (and a variety of cross breeds).

### Gene expression associated with mast cell tumour metastasis

Three-quarters of the genes that displayed statistically significant differences in expression between M and NM MCTs showed decreased expression in the M MCTs. This suggests that the ‘balance’ in the molecular genetic contribution to MCT metastasis lies in a reduction of the effect of genes that would otherwise diminish the propensity for metastatic dissemination. Functional annotation enrichment analysis of the differentially expressed genes provides an insight into the biological processes associated with MCT metastasis.

#### Cell adhesion

The loss of normal cell polarity and adhesion are pivotal to tumour metastasis. Four genes (*SCRIB*, *PKP1*, *CDSN* and *DSP*) with ‘cell adhesion’ annotation show decreased expression in M MCTs. Failure to maintain the three-dimensional organization of tissues is coincident with disruption of intercellular junctions, loss of cell adhesion and epithelial-mesenchymal transition. The scaffold protein Scribbled Planar Cell Polarity Protein (*SCRIB*) regulates cell polarity and cell proliferation. In *Drosophila*, deletion of *SCRIB* causes loss of apical-basal polarity and in concert with oncogenic *Ras* activation induces cell proliferation and metastasis [42]. Plakophilin 1 (*PKP1*), Desmoplankin (*DSP*) and corneodesmosin (*CDSN*) are components of the desmosome, intracellular junctions that link cytoskeletal intermediate filaments to the plasma membrane and mediate cell-cell adhesion. Decreased expression of *PKP1* in oral squamous cell carcinoma cells increased cell motility [43], and is associated with the metastatic phenotype of several human cancers [44]. Reduced expression of DSP in a number of human primary tumours has been associated with tumour metastasis [45]. *DSP* has also been identified as a migration suppressor in a mouse model of pancreatic cancer [46], potentially effected by inhibition of β-catenin-dependent Wnt signalling [47].

#### Canonical Wnt signalling

The role of canonical Wnt signalling in tumour metastasis is tissue-specific, and both activation and inactivation of the pathway have been associated with promoting epithelial-mesenchymal transition [48]. Herein, it is unclear whether activation or inhibition of Wnt signalling promotes MCT metastasis. Decreased expression in the M MCTs of several genes associated with canonical Wnt signalling suggests that diminution of canonical Wnt signalling promotes MCT metastasis. The transcriptional co-activator *BCL9L*, which promotes β-catenin activity and transcription of Wnt target genes, shows decreased expression in the M MCTs. A similarly reduced level of expression in the M MCTs is noted for the transcription factor, and proto-oncogene, *MYC*, a target of Wnt signalling. This result is conceptually consistent with the observation that *MYC* overexpression inhibits cancer cell motility and invasiveness *in vitro* [49]. *SDC1*, a cell surface heparan sulphate proteoglycan, promotes canonical Wnt signalling in metastatic melanoma [50]. *SDC1* links the cytoskeleton to the extracellular matrix (ECM) and has a role in cell-cell and cell-ECM adhesion, and cell migration. *SDC1* expression varies between cancer types, but reduced expression in carcinomas is associated with enhanced cell motility and invasion [51]. *PLPP3*, a membrane glycoprotein, also shows a decreased level of expression in M MCTs. A major function of *PLPP3* is dephosphorylation of extracellular lysophosphatidic acid, a phospholipid with growth factor-like activity that stimulates tumour cell migration and invasion [52]. *PLPP3* displays reduced expression in metastasising (versus non-metastasising) sporadic colorectal cancer [53]. Conversely, *SFPR4*, a member of the secreted frizzled-related family that inhibit Wnt signalling by binding to Wnt proteins or to Frizzled receptors, displays decreased expression in M MCTs.

#### Negative regulators of cell proliferation

De-regulation of cell proliferation (and a consequent high proliferation rate) is generally associated with tumour aggressiveness. Although the paradigm has been recently challenged, metastatic potential has also been associated with increased resistance/decreased sensitivity to apoptosis [54]. Negative regulators of cell proliferation are enriched amongst the genes exhibiting differential expression between M and NM MCTs. DnaJ Heat Shock Protein Family Member A3 (*DNAJA3*) is the only one of 9 genes to display increased expression in the M MCTs. *DNAJA3* encodes two protein isoforms localised to the mitochondrial matrix which have opposite effects on apoptosis induced by external stimuli. The short isoform suppresses apoptosis [55], and its overexpression has been shown to promote the migration and invasion of non-small cell lung carcinoma cells *in vitro* [56]. The genes expressed at a lower level in the M MCTs include the transcription factor *TFAP2A* and transcriptional repressors *ETV3*, *HDAC4*, and *SPRY1*.

#### Bleb assembly and cell death

The migration of individual tumour cells is facilitated by the formation of bleb plasma membrane protrusions [57], which are initially devoid of the polymeric form of actin. Peripheral myelin protein 22 (*PMP22*) is an integral membrane protein known to be localised to epithelial and endothelial cell-cell junctions. *PMP22* is involved in the linkage of the actin cytoskeleton to the plasma membrane, and overexpression of *PMP22* reduces cell growth and motility [58]. Membrane blebbing is also a hallmark of apoptosis and overexpression of *PMP22* and the epithelial membrane proteins (EMP) 1 and 2 has been shown to increase cell death *in vitro* [59]. *PMP22*, *EMP1* and *EMP2* had reduced levels of expression in M MCTs.

#### Stress fibre assembly

Four genes (*EVL*, *BRAF*, *ARHGEF10* and *NF2*) annotated as positive regulators of actin stress fibre assembly displayed decreased expression in the M MCTs. Cell softening is necessary for cell invasion and this is achieved through reorganisation of the actin cytoskeletal architecture. Ena/VASP-like (*EVL*) enhances actin polymerisation and suppresses cell migration [60]. Reduced expression of *BRAF* in mouse embryonic fibroblasts was associated with a reduction in actin stress fibre content and an increase in cell migration [61]. Rho GTPase guanine nucleotide exchange (*ARHGEF10*) activates several Rho GTPases promoting actin stress fibre formation [62]. However, transient expression of ARHGEF10 *in vitro* was associated with the loss of actin stress fibres and the formation of membrane filopodia [63], which facilitate individual tumour cell migration. Neurofibromin 2 (*NF2*) is thought to encode a protein that links components of the cytoskeletion, including actin, with plasma membrane proteins. *NF2* has been shown to stop cell migration by preventing cleavage of the actin-linked transmembrane protein CD44 [64].

#### cAMP signalling

Altered cyclic nucleotide signalling is a trait of many cancers, although the effect of signalling on cell growth and survival is cancer and cell-type dependent. Five genes associated with cAMP signalling (*CALM3*, *CREBBP*, *FOS*, *BRAF*, *AFDN*) show differential expression between M and NM MCTs. Up-regulated in the M MCTs is *CALM3*, an enzymatic co-factor involved in the regulation of adenyl cyclase (AC) through calcium signalling. Although AC generates cAMP from ATP, its intracellular level is also dependent upon phosphodiesterases. *CREBBP* binding enhances the transcription factor activity of the cAMP-response element binding protein (CREB) once it is phosphorylated by cAMP-activated protein kinase A. *CREB* mediates transcription of *FOS* and *JUN*, and homodimers of each, or heterodimers of both, form the AP-1 transcription factor complex, which regulates the expression of genes involved in proliferation, apoptosis and cell migration [65]. *EPAC*, an exchange protein activated by cAMP, activates the GTPase Ras-associated protein 1, which in turn activates *BRAF* and the adherens junction formation factor (*AFDN*). *AFDN* links nectins (transmembrane cell adhesion molecules) at cell-cell junctions to the actin cytoskeleton. The decrease in the expression of both *JUNB* and *FOS* observed in the M MCTs is consistent with the decreased expression of *CREBBP*, and may suppress diminution of proliferation because *JUNB* is typically a negative regulator of cell proliferation [66]. Down-regulation of *JUNB* in tumour metastases (relative to primary tumours) is common to many human cancers [17]. Reduced M MCT expression of *BRAF* and *AFDN* disrupts cell-cell adhesion favouring cell migration.

#### Genes displaying two-fold or greater differences in expression between M and NM MCTs

Seven of the 18 genes which show ≥two-fold decreased expression in the metastasising MCTs are keratin genes, or keratin/keratinocyte-associated genes. Keratins are intermediate filaments that form part of the cytoskeleton, and are largely associated with maintaining the mechanical stability and integrity of epithelial cells [67]. Skin epidermal tissue was estimated to constitute 2–3% of the longitudinal MCT biopsy cross-sections from which RNA was isolated for gene expression analysis, and there was no apparent gross difference between the epidermal tissue content of M and NM primary MCT biopsies. However, it is unclear whether differential expression of genes encoding keratin intermediate filaments and epithelial cell-associated desmosomal proteins (*DSP*, *PKP1*) reflects differences in epithelial cell (keratinocyte) and/or mast cell gene expression.

Keratins are not detected in canine MCTs by anti-pan cytokeratin immunohistochemistry, which screens for a number of keratins common to many epithelial tissues. However, keratin gene expression is tissue-, differentiation state and functional status-specific [67], and keratin genes have been shown to be expressed in haematopoetic cells [68], as has *DSP* [69].

In epithelial tumours the down-regulation of specific keratins is believe to alter the cytoskeleton architecture causing increased cellular elasticity and deformability such that cells are better able to permeate through the stroma and migrate away from the primary tumour [70]. It is possible that down-regulation of specific keratin genes in neoplastic mast cells has a similar effect to that deduced for epithelial tumour cells. The altered expression of 8 skin epithelial cell-associated genes (*KRT1*, *KRT5*, *KRT15*, *KRTDAP*, *DSP*, *PKP1*, *PERP* and *SBSN*) that displayed >two-fold decreased expression in the M MCTs have previously been associated with the metastasis of a non-epithelial tumour as they were expressed at lower levels in human metastatic cutaneous melanomas than in primary tumours [71]. Down-regulation of *KRT15* in tumour metastases (relative to primary tumours) is also common to many solid human cancers [17].

If keratin and desmosomal protein-encoding genes are not expressed in neoplastic canine mast cells, a possibly unlikely alternative proposition is that cytoskeletal reorganisation and reduced adhesiveness of adjacent keratinocytes (potentially neoplastic mast cell-directed) assists neoplastic mast cell cells to escape from the primary tumour. *KRT5* and *KRT15* are found in keratinocytes occupying the basal layer of the epidermis [67], and their reduced expression in the M MCTs may indicate a loss of basal epithelial cells and/or invasion of the basal layer as an early step in the metastatic cascade. There is increasing appreciation of the role of the tumour tissue microenvironment in facilitating various stages of the metastatic cascade, and evidence that the cells in a primary tumour exploit interactions with surrounding non-malignant cells and the ECM to enable inappropriate growth, local invasion and metastatic dissemination [72]. As a potential precedent, interaction between keratinocytes and cutaneous melanoma cells has been shown to be required for vertical invasion of melanoma cells into the dermis [73].

PERP (TP53 Apoptosis Effector) is a transmembrane 4 desmosomal protein that is involved in maintaining epithelial cell integrity by promoting desmosomal-mediated cell adhesion, but its transcription is also activated by p53 to effect apoptosis [74]. PERP expression is reduced in human primary uveal melanomas that metastasise [75], and PERP has been shown to be down-regulated in murine bone marrow-derived mast cells overexpressing microRNA miR-9, which displays increased expression in ‘biologically high grade MCTs’ [76]. Over-expression of miR-9 enhanced the invasion of mouse malignant mast cell cells *in vitro* [76].

SBSN (suprabasin) is located in epithelial suprabasal layers and is involved in epidermal differentiation. Both up-regulation [77] and down-regulation [71] of its expression have been associated with tumour metastasis.

SFN (Stratifin or 14-3-3 Sigma) is primarily recognised as a cell cycle check point protein which mediates cycle arrest following DNA damage. However, the identification of SFN-interacting proteins suggests a possible role for SFN in the regulation of cell adhesion, polarity and migration [78]. SFN is frequently silenced by hypermethylation in human cancers, and its decreased expression has been associated with the metastasis of several human cancers [71, 79].

PSORS1C2 (also known as SPR1) is a component of the cross-linked envelope formed on the intracellular side of the cell membrane of terminally differentiated squamous epithelial cells. Reduced PSORS1C2 expression disrupts terminal differentiation and is associated with malignant transformation [80].

CLEC3B encodes a C-type lectin (tetranectin) which is located in the ECM and binds to plasminogen in the presence of plasminogen activators to generate an active protease (plasmin). Plasmin participates in ECM and basement membrane degradation/remodelling, processes key to invasion and metastasis. A reduced serum/plasma CLEC3B level is a biomarker for the metastasis of several human cancers [81].

The role of the transcription factor EGR1 in tumour development and progression is dependent upon the sum of the functions of the genes that it regulates, but it has been shown to up-regulate multiple tumour suppressor genes to inhibit cell growth, proliferation and metastasis [82]. In certain tumour types EGR1 represses transcription of heparanase, which degrades heparan sulphate proteoglycan chains present in the ECM and basement membranes allowing tumour cells to spread and inducing the release of pro-angiogenic chemokines and growth factors [83]. Increased expression of EGR1 in human non-small cell lung carcinomas is associated with up-regulation of KRT18 and reduced lymph node metastasis [84].

CD9 (motility-related protein-1), a member of the transmembrane 4 (tetraspanin) superfamily of cell surface proteins, interacts with intergrin cell adhesion molecules, signalling proteins, and immunoglobulin superfamily members promoting adherence to the ECM and suppressing motility [85]. Decreased expression of CD9 in several human tumours is associated with increased metastatic potential [86]. Canine mast cell tumours are often considered to be an analogue of human gastrointestinal stromal tumours (GISTs) because activating mutations in *c-kit* occur in both. CD9 expression is recognised as a prognostic marker for gastric GIST [87].

The only gene to show >two-fold increased expression in the MCTs was the non-coding small nucleolar RNA C/D box 93 (SNORD93). Small nucleolar RNAs (snoRNAs) guide sequence-specific post-transcriptional modification of rRNAs and small nuclear RNAs. However, a large proportion of snoRNAs are processed into smaller small nucleolar RNA-derived RNAs (sdRNAs), and a number of C/D box-derived sdRNAs have been shown to suppress gene expression in a manner analogous to microRNAs [88]. SNORD93 has been shown to display increased expression in a metastatic breast cancer cell line [89], and a sdRNA derived from SNORD93 was shown to promote human breast cancer cell invasiveness [90]. A second epigenetic regulator of tumour metastasis, stem-loop pre-microRNA cfa-mir-632, displays increased expression in the M MCTs (M/NM = 1.83). MiR-632 is expressed at high levels in invasive and metastatic human breast cancer cells, and has been shown to down-regulate expression of the heat shock protein DNAJB6 resulting in increased invasive capabilities [91]. MicroRNA-effected gene silencing has been shown to be pivotal in regulating cell adhesion [92].

### Differentially expressed genes as targets for anti-metastasis therapeutics

Genes whose altered expression in M MCTs is pro-metastatic may constitute targets for anti-MCT metastasis therapeutics. By way of example, CD9 was the focus of a proof-of-principle study to assess the efficacy of a gene therapy approach to counter lung cancer metastasis. Adenoviral transduction of CD9 in an orthotopic lung cancer model was shown to significantly inhibit lymph node metastasis [93]. Particularly pertinent, is a potential new paradigm in anti-metastatics development that targets actin polymerisation and contractility [94], elements that are integral to both single cell and collective invasion modes of tumour cell migration [57]. Repeated reorganisation of the actin cytoskeleton and the formation of actin-based protrusions are integral to tumour cell migration strategies. In this context, the demonstration herein of the relevance of the altered expression of genes involved in the regulation of actin stress fibre assembly (*EVL*, *BRAF*, *ARHGEF10* and *NF2*), and the linkage of the actin cytoskeleton to the plasma membrane (*PMP22*) and to nectin cell adhesion molecules at cell-cell junctions (*AFDN*), respectively, to MCT metastasis is significant. If metastasis-promoting down-regulation of gene expression in M MCTs is achieved at least in part though sdRNA/microRNA-effected suppression (as the data obtained in this study suggests), microRNA inhibitors represent a potential therapeutic option [95].

### Metastasis-associated gene expression for potential classification of MCTs as metastasising or non-metastasising

The 19 genes that display >two-fold differences in expression between M and NM MCTs collectively represent a cross-breed metastasis-associated gene expression signature that could potentially be used to delineate M and NM MCTs through linear discriminant analysis. Preliminary evaluation, by cross-validation, estimated classification accuracies as 90–100% for M MCTs and 70–100% for NM MCTs. The differential expression of 9 of the 19 genes, including 2 whose identities are currently unconfirmed, was validated by RT-qPCR analysis. If the performance of the discriminator at delineating M from NM MCTs is subsequently validated through trial in further retrospective and prospective studies it would represent a uniquely objective and quantitative tool for predicting canine cutaneous MCT metastasis.

Where they are used, proliferation markers are typically deployed in combination with histological grading to predict the survival of dogs with mast cell tumour. Applying a cut-off score of 1.8, Ki-67 score is a significant predictor of survival of dogs with Patnaik grade II MCTs [11]. However, the effect of inter-operator variability in digital image capture and cell counting is unclear, and ‘poor survival’ may not be associated with metastatic disease.

A previous study sought to identify gene expression markers that are predictive of canine cutaneous MCT behaviour [23]. Gene expression in 13 Kiupel low grade tumours was compared with that in 5 Kiupel high grade tumours. Nearest shrunken centroid classification identified 13 genes that were capable of segregating MCTs into ‘differentiated’ and ‘undifferentiated’ MCT groups, although tumours from dogs that experienced MCT-related death were included in each group. In a subsequent study [96], the gene expression profiles of 40 ‘non-aggressive’ MCTs were compared with those of 7 ‘aggressive’ MCTs. However, aggressive MCTs were not selected due to evidence of distant and/or lymph node metastasis, but based on their histology, and because they were borne by dogs that received systemic treatment and survived for a certain unspecified period of time.

Unbalanced chromosomal abnormalities represent one of the mechanisms by which metastasis-associated changes in gene expression may be effected. Chromosomal grouping (on CFA1 and CFA31, respectively) of genes displaying decreased expression in the M MCTs may be indicative of focal deletions in the M MCTs and/or co-ordinated regulation of transcription. The potential for prognostically-relevant molecular classification of canine MCTs based upon copy number aberrations (CNAs) in MCTs has recently been investigated [97, 98]. CNAs were more frequent in tumours from 6 dogs that died within 6 months of diagnosis (although only 4 of the dogs had confirmed metastasis at diagnosis), and specific gene losses (*PTEN* and *FAS*; CFA26) and gains (*MAPK3*, *WNT5B*, *FGF*, *FOXM1* and *RAD51*; CFA27) were associated with a shorter survival time [97]. One of two genes on CFA26 that showed decreased expression in the M MCTs in the present study is located in a ≥1.2Mb CFA26 fragment affected by loss in ~50% of the MCTs from dogs that died within 6 months of diagnosis [97]. A second CNA profiling study identified 4 CNAs that predicted ‘high risk MCTs’ with a sensitivity of 78–94% and specificity of 88–93% [98]. Loss of one copy of CFA5 was reported in ~50% of the ‘high risk MCTs’ [98], and in the present study 15 of 16 differentially expressed genes located on CFA5 show decreased expression in the metastasising MCTs. High risk MCTs were defined as those designated as a Kiupel high grade tumour [8] and/or containing an ITD in *c-kit* exon 11 [98]. However, since neither the binary grading system nor the presence of an ITD in *c-kit* exon predicts cutaneous MCT metastasis, the prognostic utility of the proposed 4 CNA-based classification is uncertain.

### Limitations

This study featured FFPE biopsies of canine cutaneous MCTs that were surgically removed at first opinion veterinary practices from dogs that were subsequently referred to a specialist veterinary oncology centre. FFPE MCT biopsies were used because it is difficult to collect (in a referral setting) sufficient numbers of fresh (flash frozen or RNAlater-preserved) biopsies, whilst in a primary setting it is more difficult to collate definitive evidence of MCT tumour metastasis. However, global GEP of FFPE tissues using Affymetrix microarrays has been shown to yield biologically authentic and clinically-relevant data [99]. Further retrospective and prospective studies using new, larger cohorts of M and NM MCTs, optimally collected as fresh specimens, will be necessary to validate the capability of the 19-gene LDA classifier at predicting whether a canine cutaneous MCT is a metastasising or non-metastasising tumour. *In vitro* experimental investigations will ultimately be required to demonstrate if the effects (e.g. on cell adhesion, deformability and motility) on neoplastic canine mast cells of the differences in gene expression (between M and NM MCTs) observed in the current study are as is predicted based on what is known about the function(s) of the genes concerned and (in some cases) data from previous *in vitro* studies.

### Conclusions

Changes in gene expression that mediate metastasis may be temporal, effected by similarly temporal epigenetic regulation, and/or may reflect somatic alterations that become advantageous or are newly acquired in migrating tumour cells. The differences in gene expression displayed by the primary cutaneous mast cell tumours that metastasised (relative to those that did not) appear to reflect the requirements of the initial ‘invasion phase’ of the metastatic cascade. Cell migration is facilitated by loss of cell polarity, reduced cell-cell and cell-ECM adhesion, and cell softening achieved through cytoskeletal reorganisation and disruption of cytoskeleton-plasma membrane links. De-regulation of cell proliferation, and suppression of cell cycle arrest and apoptosis, support invasion, whilst a pro-angiogenic reduction in EGR1 expression promotes intravasation. Some of the genes whose altered expression mediates canine cutaneous MCT metastasis may be potential targets for anti-MCT metastasis therapeutics. This will depend upon the mechanism by which gene expression is altered, and the specificity of the biological function(s) of the genes concerned. Measurement of the expression levels of the 19 genes that display greater than two-fold differences in expression between M and NM primary MCTs may have the potential to form the basis of a test that will predict with a high degree of certainty whether a cutaneous MCT will metastasise. If the performance of the metastasis gene expression signature-associated LDA classifier is validated on an independent MCT cohort it would represent the only test for canine cutaneous MCT metastasis and, as a single assay, an improvement on currently available prognostic indicators for canine cutaneous MCTs.

## Supporting information

S1 File Supporting information. (PDF)

S1 Fig Unsupervised hierarchical clustering of 34 MCTs on the basis of the expression values of the 20% of Transcript clusters (1,041) with the highest variance in expression signal. (A) MCTs labelled according to breed: B—Boxer; CB—Cross breed, CCR—Curly Coated Retriever, D—Dogue de Bordeaux, ETT—English Toy Terrier, GS- German Shepherd, HV—Hungarian Vizsla, LR—Labrador Retriever, MS—Miniature Schnauzer, SBT—Staffordshire Bull Terrier, W—Whippet. (B) MCTs labelled according to sex: Fe—Female, FeN—Neutered female, Ma—Male, MaN—Neutered male. (C) MCTs labelled according to age: Numbers indicate age in years.
(PDF)

S1 Table Reagents used in quantitative PCR assays. (A) RNA integrity assay. (B) Assay for reverse transcription and/or PCR inhibitors. (C) RT-qPCR assays for quantitation of gene expression (optimal probe and primer concentrations, established experimentally, are listed).
(PDF)

S2 Table Assay for reverse transcription and PCR inhibitors in MCT RNAs. (A) Metastasising MCTs. (B) Non-metastasising MCTs.
(PDF)

S3 Table Primary cutaneous mast cell tumour RNAs used for gene expression profiling. (A) Metastasising MCTs. (B) Non-metastasising MCTs.
(PDF)

S4 Table RNA sample quality metrics for identification of ‘outlier’ arrays. (PDF)

S5 Table Chromosomal locations of 216 genes that are differentially expressed (permutation-testing-adjusted p value <0.05) between M and NM MCTs. (A) Proportion of genes expressed in MCTs that are located on each chromosome. (B) Positions of differentially expressed genes on chromosome 31.
(PDF)
