# Supplementary material for: Identification of molecular genetic contributants to canine cutaneous mast cell tumour metastasis by global gene expression analysis
Source: PLoS One. 2018 Dec 19;13(12):e0208026. doi: 10.1371/journal.pone.0208026 (PMC6300220; doi:10.1371/journal.pone.0208026)
Supplement: S1 File — (PDF) [file pone.0208026.s001.pdf]

S1 File. Supporting Information

Materials and Methods

Assay of RNA integrity

The integrity of each formalin-fixed, paraffin-embedded (FFPE) mast cell tumour (MCT) RNA sample was assessed by reverse transcription-quantitative PCR (RT-qPCR) assay of a 126bp fragment of a 130 - 150bp short interspersed nuclear element (SINE) present every 5 - 8.3kb in the canine genome [1]. Each MCT cDNA (18µl) was diluted with 10µl of 1mM Tris-HCl (pH 8.0), 0.1mM EDTA and 1µl used in triplicate 10µl PCR reactions, comprising 1 x PowerUp SYBR Green Master Mix (ThermoFisher Scientific, Paisley, UK) and 0.3µM of each of the CfSINE126 PCR primers (S1 Table). The PCR reaction master mix was UV irradiated at 302nm for 5 min prior to addition of the CfSINE126 PCR primers. Thermocycling (ABI StepOne Plus; ThermoFisher Scientific, Paisley, UK) was performed as follows: 50°C, 2 min; 95°C, 2 min; (95°C, 3s; 60°C, 30s) x 40; melt curve program.

Reverse transcription-quantitative PCR (RT-qPCR)

Reverse transcription

cDNA was prepared (in a 20µl reaction) from 111ng of each MCT RNA using the High-Capacity cDNA Reverse Transcription Kit (ThermoFisher Scientific, Paisley, UK).

Assay for reverse transcription and PCR inhibitors

Prior to use in RT-qPCR assays, each MCT cDNA was screened for the presence of PCR (and potentially reverse transcription) inhibitors. An aliquot of each MCT cDNA was spiked with an equal amount of a synthetic *solanum tuberosum* (SPUD)-derived amplicon [2], and the SPUD amplicon quantification cycle (Cq) value obtained for each upon PCR amplification compared with that measured for the SPUD amplicon in the absence of MCT cDNA. Duplicate 10µl PCR reactions were performed for each MCT cDNA, and comprised 1.5µl of cDNA, 1 x PowerUp SYBR Green Master Mix (ThermoFisher Scientific, Paisley, UK), 1.33fM of SPUD amplicon (S1 Table), and 0.3µM of forward and reverse SPUD primers (S1 Table). In duplicate 'SPUD amplicon alone reactions', an MCT cDNA was replaced with 1.5µl of nuclease-free water. PCR reactions were run in an ABI StepOne Plus machine (ThermoFisher Scientific) using the following program: 98°C, 2min; (98°C, 5s; 60°C, 30s) x 40; melt curve program.

cDNA preamplification

An 11µl aliquot of each MCT cDNA was preamplified in a 50µl reaction, comprising 1 x TaqMan PreAmp Master Mix (ThermoFisher Scientific, Paisley, UK) and 45nM of both the forward and reverse PCR primer (S1 Table) for each gene to be subsequently assayed. Preamplification was performed on a DNA Engine Tetrad (Bio-Rad) at 95°C for 10 min, followed by 14 cycles of (95°C for 15s and 60°C for 4 min). A 45µl aliquot of each preamplified cDNA was diluted with 855µl of nuclease-free water, and 2.5µl aliquots were used for quantitative PCR.

Quantitative PCR

Triplicate PCR reactions were performed for each preamplified MCT cDNA. TaqMan PCR reactions (10µl) comprised 1 x TaqMan Gene Expression Master Mix (ThermoFisher Scientific, Paisley, UK) and the TaqMan assay reagents (S1 Table). Thermocycling (ABI StepOne Plus; ThermoFisher Scientific, Paisley, UK) was performed as follows: 50°C, 2 min; 95°C, 10 min; (95°C, 15s; 60°C, 1 min) x 40. SYBR Green PCR reactions (10µl), comprising 1 x PowerUp SYBR Green Master Mix (ThermoFisher Scientific, Paisley, UK) and forward and reverse PCR primers (S1 Table), were thermocycled (ABI StepOne Plus; ThermoFisher Scientific, Paisley, UK) as follows: 50°C, 2 min; 95°C, 2 min; (95°C, 3s; 60°C, 30s) x 40; melt curve program.

Results

Microarray data analysis

Identification of ‘outlier arrays’

Six arrays (CB7, JRT1, LR11, ESS1, MS2 and P1) that had one ‘RNA sample quality metric’ [3] 2 standard deviations away from the mean of the metric value for the sample cohort were flagged as outliers [4] (S4 Table) and excluded.

Reverse transcription-quantitative PCR (RT-qPCR)

Assay for reverse transcription and PCR inhibitors

All the MCT RNAs (with the exception of LR6) had a higher mean Cq than the SPUD amplicon alone (median difference = +0.39 cycles for M MCTs and +0.42 cycles for the NM MCTs; S2 Table). However, the extent of the putative inhibitor-related increase in the SPUD amplicon Cq values measured in the presence of the MCT RNAs is similar for both the M and NM MCTs (the median M and NM MCT group Cq values differ by 0.03, and the mean M and NM MCT group Cq values differ by 0.02) (S2 Table). Differences of this magnitude will be adequately adjusted for by normalisation when generating relative expression values. Furthermore, the differences between the SPUD amplicon Cq values measured in the presence and absence of a MCT RNA (<0.5

cycles, which is often used as the permissible Cq standard deviation for replicate qPCR assays) do not suggest that inhibition of reverse transcription and/or PCR would significantly affect RT-qPCR assay sensitivity.

References

1. Das M, Chu LL, Ghahremani M, Abrams-Ogg T, Roy MS, Housman D, et al. Characterization of an abundant short interspersed nuclear element (SINE) present in Canis familiaris. Mamm Genome. 1998;9:64-69.
2. Nolan T, Hands RE, Ogunkolade W, Bustin SA. SPUD: a quantitative PCR assay for the detection of inhibitors in nucleic acid preparations. Anal Biochem. 2006;351:308-310.
3. Affymetrix White Paper: Quality Assessment of Exon and Gene Arrays. Revision Date: 2007-04-06, Revision Version: 1.1. Available from: https://assets.thermofisher.com/TFS-Assets/LSG/brochures/exon_gene_arrays_qa_whitepaper.pdf.
4. QC Metrics for Exon and Gene Design Expression Arrays. A summary based on the Affymetrix Quality Assessment of Exon and Gene Arrays White Paper. Available from: http://static1.1.sqspcdn.com/static/f/1438485/21486054/1359060361517/qc_metrics_exon_gene_qrc.pdf.
