# Supplementary material for: Identification of molecular genetic contributants to canine cutaneous mast cell tumour metastasis by global gene expression analysis
Source: PLoS One. 2018 Dec 19;13(12):e0208026. doi: 10.1371/journal.pone.0208026 (PMC6300220; doi:10.1371/journal.pone.0208026)
Supplement: S1 Fig — (A) MCTs labelled according to breed: B—Boxer; CB—Cross breed, CCR—Curly Coated Retriever, D—Dogue de Bordeaux, ETT—English Toy Terrier, GS- German Shepherd, HV—Hungarian Vizsla, LR—Labrador Retriever, MS—Miniature Schnauzer, SBT—Staffordshire Bull Terrier, W—Whippet. (B) MCTs labelled according to sex: Fe—Female, FeN—Neutered female, Ma—Male, MaN—Neutered male. (C) MCTs labelled according to age: Numbers indicate age in years. (PDF) [file pone.0208026.s002.pdf]

Figure S1. Unsupervised hierarchical clustering of 34 MCTs on the basis of the expression values of the 20% of Transcript clusters (1,041) with the highest variance in expression signal

A. MCTs labelled according to breed.

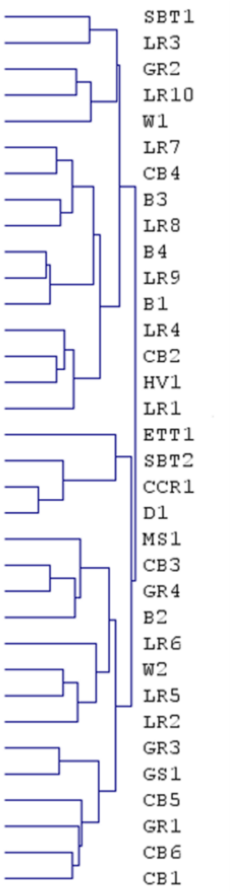

B - Boxer; CB - Cross breed, CCR - Curly Coated Retriever, D - Dogue de Bordeaux, ETT - English Toy Terrier, GS- German Shepherd, HV - Hungarian Vizsla, LR - Labrador Retriever, MS - Miniature Schnauzer, SBT - Staffordshire Bull Terrier, W - Whippet.

B. MCTs labelled according to sex.

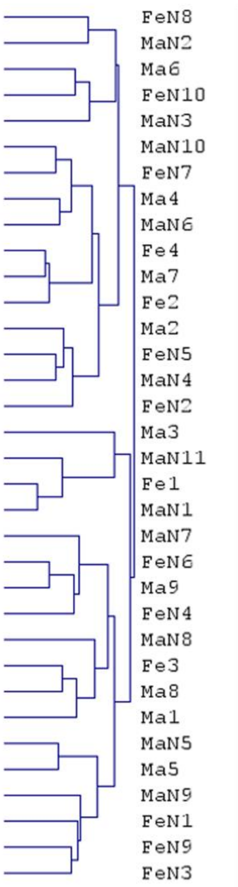

Fe - Female, FeN - Neutered female, Ma - Male, MaN - Neutered male.

C. MCTs labelled according to age.

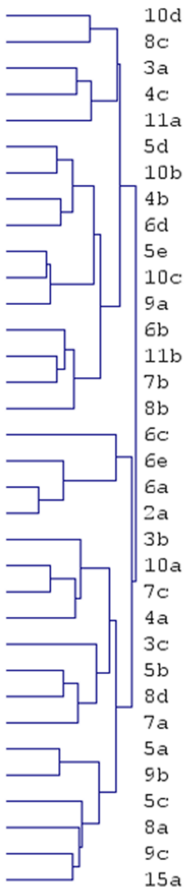

Numbers indicate age in years.
