# Supplementary material for: Identification of molecular genetic contributants to canine cutaneous mast cell tumour metastasis by global gene expression analysis
Source: PLoS One. 2018 Dec 19;13(12):e0208026. doi: 10.1371/journal.pone.0208026 (PMC6300220; doi:10.1371/journal.pone.0208026)
Supplement: S1 Table — (A) RNA integrity assay. (B) Assay for reverse transcription and/or PCR inhibitors. (C) RT-qPCR assays for quantitation of gene expression (optimal probe and primer concentrations, established experimentally, are listed). (PDF) [file pone.0208026.s003.pdf]

S1 Table. Reagents used in quantitative PCR assays.

A. RNA integrity assay.

**Gene ID.**	**Forward primer sequence** (5'-3')	**Reverse primer sequence** (5'-3')	**PCR product size** (bp)
CfSINE126	GATCCCTGGGTGGCGCA	GAGACACAGGCAGAGGGAGA	126

B. Assay for reverse transcription and/or PCR inhibitors.

**ID.**	**Sequence** (5'-3')
SPUD Amplicon	AACTTGGCTTTAATGGACCTCCAATTTTGAGTGTGCACAAGCTATGGAACACCACGTAAGACATAAAACGGCCACATATGGTGCC ATGTAAGGATGAATGT
SPUD Forward Primer	AACTTGGCTTTAATGGACCTCCA
SPUD Reverse Primer	ACATTCATCCTTACATGGCACCA

C. RT-qPCR assays for quantitation of gene expression (optimal probe and primer concentrations, established experimentally, are listed

TaqMan assays

	**Probe**		**Forward Primer**		**Reverse Primer**			
**Gene ID.**	**Sequence** (5'-3')	**Conc.** (nM)	**Sequence** (5'-3')	**Conc.** (nM)	**Sequence** (5'-3')	**Conc.** (nM)	**PCR product size** (bp)	**Reaction efficiency** (%)
KRT10	TTGAACAAATGTCCAGCCACAAATCTG	250	CTGACTACAGAAATCAACAG	900	GCGTCTCAATTCAGTAATC	300	72	71.51
SBSN	ACCGAGGGCTGACCAATGCA	150	GAAGGTCATTGAAGGAATC	900	GAGTGATTCCATTATTGATGC	900	89	84.63
EGR1	CCACCTCCTATCCGTCACCTGTA	50	CTACCTCTTATCCCTCCC	300	ATGGGTAGGTTGAGGAA	300	87	81.16
DSP	AGCAGATCCACTCCCAGACTTCA	150	CCATGAAGACGGAACTG	300	TCCAGGTCATAGAGAGG	900	76	119.73
PERP	TCTTTCTTTGCCCTCTGCGGAC	150	TGGTGATCTGCTTCATC	300	CCTCCAATCACTCTCAG	900	76	82.84
CD9	CAAGACACCCAGCCTTCTAGCTTC	50	GACCAAGAGCATCTTTGA	900	CTCCAATCAGAATATAAACTCC	900	71	105.91

SYBR Green assays

	Forward Primer		Reverse Primer			
**Gene ID.**	**Sequence** (5'-3')	**Conc.** (nM)	**Sequence** (5'-3')	**Conc.** (nM)	**PCR product size** (bp)	**Reaction efficiency** (%)
SNORD93	GCCGAGGATGAGAAATCTAATCT	300	AACGCCATCAGAGAAGCATAT	300	50	99.64
ENSCAFG00000030560	TTCCCTGCTGGAGACAA	800	GGATGATCTGGATCCTTCTG	800	53	102.26
KRTAP4-4	TTGTGGCTCCGTCTGCTCTG	500	CGGCAGCAGGTCTCCTCG	800	51	100.29
CfSINE71*	GGAGACCCGGGATCGAAT	300	GAGACACAGGCAGAGGGAG	300	71	124.22

*Reference gene for calculation of relative gene expression levels
