# Supplementary material for: Identification of molecular genetic contributants to canine cutaneous mast cell tumour metastasis by global gene expression analysis
Source: PLoS One. 2018 Dec 19;13(12):e0208026. doi: 10.1371/journal.pone.0208026 (PMC6300220; doi:10.1371/journal.pone.0208026)
Supplement: S2 Table — (A) Metastasising MCTs. (B) Non-metastasising MCTs. (PDF) [file pone.0208026.s004.pdf]

S2 Table. Assay for reverse transcription and PCR inhibitors in MCT RNAs.

SPUD amplicon alone Cq = 25.36 ± 0.003

A. Metastasising MCTs.

Dog ID.	Mean Cq	Cq SD	Cq - SPUD Amplicon Cq
B1	25.71	0.02	0.35
CB1	25.69	0.01	0.33
CB4	25.84	0.12	0.48
CB6	25.76	0.03	0.40
CCR1	25.75	0.05	0.39
D1	25.77	0.00	0.41
GR1	25.81	0.01	0.45
GS1	25.68	0.04	0.32
HV1	25.75	0.03	0.39
LR1	25.71	0.04	0.35
LR10	25.69	0.06	0.33
LR2	25.75	0.00	0.39
LR3	25.74	0.06	0.38
LR5	25.75	0.04	0.39
LR8	25.67	0.02	0.31
LR9	25.83	0.05	0.47
SBT1	25.75	0.02	0.39
W1	25.68	0.07	0.32

Mean and standard deviation	25.74 ± 0.05		0.38 ± 0.05
Median	25.75		0.39
Interquartile range	0.06		

B. Non-metastasising MCTs.

Dog ID.	Mean Cq	Cq SD	Cq - SPUD Amplicon Cq
B2	25.88	0.06	0.52
B3	25.76	0.04	0.40
B4	25.68	0.03	0.32
CB2	25.80	0.04	0.44
CB3	25.87	0.04	0.51
CB5	25.70	0.02	0.34
ETT1	25.80	0.06	0.44
GR2	25.74	0.00	0.38
GR3	25.83	0.11	0.47
GR4	25.55	0.00	0.19
LR4	25.79	0.11	0.43
LR6	25.04	0.89	-0.32
LR7	25.80	0.09	0.44
MS1	25.77	0.03	0.41
SBT2	25.81	0.05	0.45
W2	25.74	0.07	0.38

Mean and standard deviation	25.72 ± 0.19		0.36 ± 0.19
Median	25.78		0.42
Interquartile range	0.07		
