# Supplementary material for: Identification of molecular genetic contributants to canine cutaneous mast cell tumour metastasis by global gene expression analysis
Source: PLoS One. 2018 Dec 19;13(12):e0208026. doi: 10.1371/journal.pone.0208026 (PMC6300220; doi:10.1371/journal.pone.0208026)
Supplement: S3 Table — (A) Metastasising MCTs. (B) Non-metastasising MCTs. (PDF) [file pone.0208026.s005.pdf]

S3 Table. Primary cutaneous mast cell tumour RNAs used for gene expression profiling.

A. Metastasising MCTs.

**Dog ID.**	**Age of FFPE tumour at RNA isolation** (Years)	**RNA integrity** (SINE Cq)
CB7[a]	2	21.91
CB1	5	22.46
CCR1	4	23.09
ESS1[a]	4	23.74
LR5	5	24.49
CB6	5	25.08
CB4	3	25.29
W1	4	25.44
B1	8	25.49
LR8	3	25.98
GS1	7	26.48
LR2	3	26.69
LR9	3	27.36
LR10	14	27.59
HV1	3	27.97
SBT1	3	27.99
LR3	4	28.87
GR1	2	29.18
D1	11	29.28
LR1	3	30.03

[a]'Outlier RNA samples' excluded prior to differential gene expression analysis

Prior to 'outlier array' exclusion

Mean and standard deviation	4.80 ± 2.99	26.22 ± 2.28
Median	4.00	26.23
Interquartile range	2.00	3.35

Following 'outlier array' exclusion

Mean and standard deviation	5.00 ± 3.07	26.60 ± 2.07
Median	4.00	26.59
Interquartile range	2.50	2.97

B. Non-metastasising MCTs.

**Dog ID.**	**Age of FFPE tumour at RNA isolation** (Years)	**RNA integrity** (SINE Cq)
LR6	4	21.88
CB3	9	23.46
MS2[a]	7	24.27
W2	4	24.42
GR3	8	24.55
MS1	4	24.60
LR7	3	24.89
JRT1[a]	13	25.06
B3	5	25.54
CB2	10	26.03
B2	10	26.12
B4	13	26.39
CB5	7	26.76
SBT2	10	26.86
GR2	12	27.10
P1[a]	10	27.11
LR11[a]	9	27.13
GR4	10	27.77
LR4	5	28.28
ETT1	11	29.63

[a]'Outlier RNA samples' excluded prior to differential gene expression analysis

Prior to 'outlier array' exclusion

Mean and standard deviation	8.20 ± 3.08	25.89 ± 1.76
Median	9.00	26.07
Interquartile range	5.00	2.54

Following 'outlier array' exclusion

Mean and standard deviation	7.81 ± 3.15	25.89 ± 1.86
Median	8.50	26.07
Interquartile range	5.75	2.48
