# Supplementary material for: Identification of molecular genetic contributants to canine cutaneous mast cell tumour metastasis by global gene expression analysis
Source: PLoS One. 2018 Dec 19;13(12):e0208026. doi: 10.1371/journal.pone.0208026 (PMC6300220; doi:10.1371/journal.pone.0208026)
Supplement: S4 Table — (PDF) [file pone.0208026.s006.pdf]

S4 Table. RNA sample quality metrics for identification of 'outlier' arrays.

	all_probeset_mean	all_probeset_rle_mean	all_probeset_mad_residual_mean	pos_vs_neg_auc	%P
GR1	0.05	0.16	0.09	0.23	0.38
LR1	0.26	0.86	0.72	0.41	1.40
LR2	0.08	0.09	0.07	0.08	0.94
D1	0.02	0.06	0.08	0.03	0.60
CB1	0.33	0.02	0.12	0.25	0.87
LR3	0.14	0.22	0.04	0.00	0.89
CCR1	0.24	0.08	0.20	0.31	0.82
W1	0.16	0.74	0.54	0.25	0.17
B1	0.27	0.53	0.43	0.50	1.72
HV1	0.29	0.35	0.31	0.39	1.23
CB7[a]	0.63	0.29	0.22	0.31	5.88[b]
B2	0.20	0.08	0.12	0.18	1.61
JRT1[a]	0.37	0.07	0.11	0.24	2.92[b]
CB2	0.25	0.30	0.26	0.39	1.61
LR4	0.26	0.17	0.21	0.25	1.38
ETT1	0.11	0.11	0.08	0.04	0.10
LR11[a]	0.41	0.02	0.13	0.26	2.75[b]
B3	0.19	0.02	0.10	0.22	1.50
CB4	0.18	0.05	0.04	0.17	1.41
ESS1[a]	0.30	0.23	0.30	0.28	2.55[b]
LR9	0.19	0.13	0.08	0.35	1.80
SBT1	0.15	0.05	0.09	0.20	0.47
LR8	0.19	0.18	0.17	0.33	1.40
LR5	0.03	0.35	0.18	0.03	0.43
W2	0.13	0.28	0.10	0.04	0.91
MS1	0.13	0.28	0.22	0.25	0.77
LR6	0.07	0.30	0.18	0.21	0.02
CB5	0.11	0.33	0.13	0.11	0.74
LR7	0.12	0.23	0.14	0.10	1.47
MS2[a]	0.37	0.19	0.31	0.38	2.01[b]
B4	0.18	0.32	0.24	0.41	1.81
GR4	0.14	0.20	0.02	0.06	0.77
P1[a]	0.48	0.10	0.21	0.35	3.89[b]
CB6	0.11	0.33	0.14	0.07	0.92
LR10	0.21	0.00	0.07	0.05	0.31
SBT2	0.03	0.27	0.13	0.11	0.83
GS1	0.14	0.26	0.18	0.16	1.05
CB3	0.08	0.50	0.27	0.01	0.80
GR2	0.23	0.42	0.28	0.07	0.11
GR3	0.25	0.24	0.21	0.23	0.87

[a]Outlier array and [b]associated RNA sample quality metric
