# Supplementary material for: Identification of molecular genetic contributants to canine cutaneous mast cell tumour metastasis by global gene expression analysis
Source: PLoS One. 2018 Dec 19;13(12):e0208026. doi: 10.1371/journal.pone.0208026 (PMC6300220; doi:10.1371/journal.pone.0208026)
Supplement: S5 Table — (A) Proportion of genes expressed in MCTs that are located on each chromosome. (B) Positions of differentially expressed genes on chromosome 31. (PDF) [file pone.0208026.s007.pdf]

S2 Table. Chromosomal locations of 216 genes that are differentially expressed (permutation-testing-adjusted p value <0.05) **between M and NM MCTs.**

A. Proportion of genes expressed in MCTs that are located on each chromosome.

Chromosome	Total No. of Genes expressed in MCTs	No. of Differentially Expressed Genes	p-value[a]
1	304	18	0.146
2	203	10	0.594
3	126	4	0.820
4	159	4	0.417
5	223	16	0.072
6	233	11	0.738
7	200	9	0.857
8	159	6	1.000
9	311	20	0.062
10	201	8	1.000
11	139	4	0.664
12	158	3	0.218
13	98	2	0.440
14	105	10	0.026
15	153	2	0.094
16	105	4	1.000
17	151	11	0.099
18	169	9	0.440
19	56	2	1.000
20	249	6	0.192
21	92	4	0.796
22	55	1	0.728
23	89	4	0.790
24	148	6	1.000
25	117	4	1.000
26	119	3	0.489
27	119	7	0.357
28	108	2	0.327
29	56	2	1.000
30	109	2	0.328
31	52	6	0.029
32	55	1	0.728
33	63	1	0.521
34	60	1	0.518
35	46	4	0.413
36	46	1	1.000
37	72	2	0.770
38	59	3	0.740
X	166	3	0.163

Genes represented by 29 of the Transcript clusters that are expressed in the MCTs (at least 1 exon probe set was 'present' in at least 30% of the tumours in the NM and/or M MCT cohort) are located in the mitochondrial genome. Genes represented by 44 of the Transcript clusters that are expressed in the MCTs and 2 of the Transcript clusters that are differentially expressed have not been assigned to a chromosome.

[a]The p-value (two-tailed) is the statistical significance of the association between chromosomal assignment and differential expression status as determined by the Fisher's exact test.

B. Positions of differentially expressed genes on chromosome 31.

**Gene description**	**Gene start** (bp)	**Gene end** (bp)	**Fold change**[c] (NM/M)
Keratin associated protein 8-1	25810799	25810990	1.59
Keratin associated protein 7-1	25830479	25830742	1.91
Keratin associated protein 11-1	25880031	25880492	1.65
ENSCAFG00000028769[a]	29098326	29107773	0.86
Bromodomain and WD repeat domain containing 1	34121818	34240553	1.05
14385288[b]	40746035	40804628	2.04

[a]Novel Ensembl protein coding gene.
[b]Transcript cluster with no gene annotation. The base co-ordinates of the start and end of the Transcript cluster are detailed.
[c]Ratio of median gene-level expression values.
